# The Impact of Nonsurgical Periodontal Therapy on the Salivary Proteome: A Pilot Study

**DOI:** 10.1155/ijod/6655743

**Published:** 2025-08-06

**Authors:** Stefania Bergamini, Elisa Bellei, Valentina Selleri, Roberta Salvatori, Giulia Micheloni, Milena Nasi, Marcello Pinti, Carlo Bertoldi

**Affiliations:** ^1^Department of Surgical, Medical, Dental and Morphological Sciences, University of Modena and Reggio Emilia, Modena, Italy; ^2^Department of Life Sciences, University of Modena and Reggio Emilia, Modena, Italy; ^3^Department of Maternal, Child and Adult Medical and Surgical Sciences, University of Modena and Reggio Emilia, Modena, Italy

**Keywords:** biomarkers, mass spectrometry, nonsurgical periodontal therapy, periodontitis, proteomics, saliva

## Abstract

**Background:** Periodontitis (Pd) is a prevalent chronic inflammatory disease of the periodontium, leading to progressive destruction of tooth-supporting tissues. Early diagnosis is crucial to prevent disease progression and improve therapeutic outcomes. Saliva has emerged as a promising diagnostic fluid due to its noninvasive collection and potential for biomarker discovery. This study employs proteomic analysis to identify salivary biomarkers associated with Pd and to evaluate the impact of nonsurgical periodontal therapy (NSPT) on the salivary proteome.

**Methods:** Comparative proteomic analysis was performed on saliva samples from periodontally healthy individuals (G1 group) and patients with advanced Pd (G2 group). For G2 patients, samples were collected both before and after NSPT. Mass spectrometry (MS) was used to identify differentially expressed proteins. Additionally, cell-free mitochondrial DNA (cf-mtDNA) was quantified as a marker of tissue damage and endogenous inflammation, and bacterial DNA as an indicator of microbial burden, to better characterize the inflammatory microenvironment.

**Results:** Proteomic profiling identified 66 salivary proteins differentially expressed in Pd patients at baseline compared to healthy controls, mainly associated with inflammation, immune response, oxidative stress, and extracellular matrix (ECM) degradation. These proteins significantly decreased following NSPT, correlating with improved clinical periodontal parameters. Notably, cf-mtDNA was elevated in Pd patients and decreased after treatment, mirroring the changes observed in bacterial DNA.

**Conclusions:** Salivary proteome analysis revealed a distinct disease-associated protein signature in G2 group, supporting its potential as a noninvasive tool for diagnosis and monitoring of Pd patients. The post-NSPT shifts in protein expression further highlight the effectiveness of periodontal therapy in modulating inflammatory biomarkers. Future studies on larger cohorts are needed to validate these findings and advance saliva-based proteomics toward personalized periodontal care.

## 1. Introduction

Periodontitis (Pd) is one of the most widespread chronic inflammatory diseases of the periodontium [1–3]. Its incidence peaks around the age of 40, with a prevalence exceeding 40% among lower socioeconomic classes in industrialized countries. Severe forms of Pd affect approximately 11% of the global population [1, 4, 5]. If left untreated, Pd leads to progressive and irreversible destruction of the tooth-supporting structures, ultimately resulting in tooth loss. This condition is associated with a significant decline in masticatory function, negatively impacting quality of life, compromising general health, and carrying substantial socioeconomic consequences [6, 7].

Pd is classified into four progressive stages (I–IV) to define the severity and extent of the disease. Additionally, it is categorized into three grades based on direct or indirect evidence of progression rate: slow, moderate, and rapid progression (grades A, B, and C) [8].

The pathogenesis and progression of Pd are not yet fully understood, but it is thought to result from an imbalance in homeostasis between the host and oral microbiota, leading to an inappropriate chronic inflammatory and immune response [7, 9]. However, the mere presence of an oral ecosystem does not necessarily lead to the development of Pd but rather, a combination of host-related and lifestyle factors triggers the degeneration of the oral ecosystem and initiates a chronic and inefficient inflammatory response [7, 10, 11]. Moreover, several studies have shown that Pd exacerbates the clinical profile of existing chronic conditions, such as cardiovascular disease, rheumatoid arthritis, and diabetes [12–16]. Conversely, Pd treatment has been shown to improve the clinical outcomes of these conditions [15, 16].

Cause-related therapy consists of nonsurgical periodontal therapy (NSPT), which corresponds to steps 1 and 2 of the European Federation of Periodontology (EFP) guidelines [17]. This approach is crucial in periodontal care, and NSPT has proven effective in reducing inflammation, decreasing the number of diseased sites, and eliminating most periodontal pockets [18].

Early diagnosis of Pd is essential to prevent irreversible tissue damage, halt disease progression, and facilitate the most appropriate therapeutic interventions. Identifying early and predictive Pd biomarkers through accurate and noninvasive methods is therefore crucial for developing rational treatment plans tailored to the disease stage and severity, based on objective rather than subjective screening tools.

Saliva has gained prominence as a diagnostic fluid due to its unique properties and significant potential for noninvasive disease monitoring [19]. It contains a complex mixture of biomolecules, including proteins, nucleic acids, electrolytes, and hormones, originating from both local (oral cavity) and systemic sources. Notably, 20%–30% of salivary proteins are derived from the bloodstream [20, 21]. These biomolecules provide insights into the etiopathogenesis of the disease and serve as valuable tools for diagnosing and monitoring both oral and systemic conditions [22, 23]. Furthermore, saliva represents a noninvasive, cost-effective alternative to traditional diagnostic methods [6, 24–26].

Among its diagnostic applications, the simultaneous analysis of salivary cell-free mitochondrial DNA (cf-mtDNA) and bacterial DNA enhances the evaluation of the periodontal microenvironment. Thanks to its ability to capture molecular signals from both local inflammation and systemic circulation, saliva serves as an ideal medium for this dual assessment. While bacterial DNA indicates the microbial burden, recognized as the primary etiological factor of periodontal disease, cf-mtDNA acts as an endogenous biomarker, reflecting tissue damage and inflammatory processes. This integrative approach underscores the diagnostic versatility of saliva in periodontal disease monitoring.

Additionally, in the context of periodontology, profiling the salivary proteome characteristic of Pd could improve early diagnosis, prognosis, and therapeutic decision-making [27].

This study aimed to compare the salivary proteome of periodontally healthy subjects and patients with advanced Pd, both before and after NSPT, to identify novel diagnostic and predictive markers. Proteomic analysis was performed using sodium dodecyl sulfate–polyacrylamide gel electrophoresis (SDS–PAGE) and LC–mass spectrometry (MS)/MS, alongside the quantification of cf-mtDNA and bacterial DNA, to provide an integrated overview of the inflammatory and microbial landscape in Pd.

## 2. Materials and Methods

### 2.1. Chemicals and Reagents

Dithiothreitol (DTT), 2x Laemmli sample buffer, ampholine, coomassie blue G-250, and Precision Plus Protein Standards Dual Blue, as well as protein assay dye reagent, were purchased from Bio-Rad Laboratories (Hercules, CA, USA). Urea, thiourea, iodoacetamide, Tris, bovine serum albumin (BSA), trichloroacetic acid (TCA), trifluoroacetic acid (TFA), 3-([3-cholamidopropyl]-dimethylammonio)-propane-sulfonate (CHAPS), and 2-mercaptoethanol were obtained from Merck KGaA (Darmstadt, Germany). Precast gel Bolt 4%–12% Bis-Tris Plus and MES SDS 10x running buffer were acquired from Life Technologies (Carlsbad, CA, USA). Trypsin Gold MS grade was from Promega Corporation (Madison, WI, USA). Versylene sterile water (for electrophoresis and MS protocols) was obtained from Fresenius Kabi (Bad Homburg, Germany), while acetone, glacial acetic acid, and acetonitrile (ACN), all of MS-grade purity, were supplied by Carlo Erba Reagents (Milan, Italy).

### 2.2. Enrollment of Participants

This study was conducted at the Periodontology Unit of the Dentistry and Oral-Maxillofacial Surgery Department of the Modena University Hospital. All procedures were approved and supervised by the local Ethics Committee of the Health Service of the Emilia-Romagna Region (University-Hospital of Modena, Protocol Number 3968/2017, Registration Number 315/17; Amendment Protocol AOU 0026796/22).

Periodontally healthy subjects (G1 group) and patients undergoing dental and periodontal therapy (G2 group) were enrolled between January 2023 and February 2024. Inclusion criteria comprised individuals aged 18 years or older, who were nonpregnant, nonlactating, nonsmokers, and without a history of alcohol abuse or major systemic diseases. Subjects were excluded if they presented bone disease, were taking anti-inflammatory medications, had diabetes or unstable clinical conditions, or required antibiotic prophylaxis [28, 29]. Participants were recruited during routine dental visits and underwent a thorough clinical examination, including a detailed medical history and periodontal screening. Based on these assessments, subjects were assigned to one of two groups according to their periodontal status. Group G1 consisted of periodontally healthy individuals, with no clinical signs of gingivitis or Pd, as defined by the diagnostic criteria of Tonetti et al. [8] and Trombelli et al. [30]. Group G2 included patients diagnosed with advanced periodontal disease, corresponding to stage III or IV according to the classification system proposed by Tonetti et al. [8]. All participants provided written informed consent for all study procedures, in accordance with the principles of the Declaration of Helsinki [31].

### 2.3. Dental–Periodontal Procedures and Clinical Measurements

Considering the EFP guidelines for the treatment of Pd, the study was structured in two main phases. The first phase, conducted during patient selection and referred to as time point zero (TP0), focused on patient re-education in oral hygiene practices and included an initial clinical assessment, medical history collection, and dental screening. At TP0, both periodontally healthy subjects (G1) and patients with Pd (G2) underwent a full periodontal charting and, when necessary, diagnostic-quality radiographs (G1-TP0 and G2-TP0).

All clinical measurements were carried out by an independent examiner qualified through dedicated training and experience. The periodontal evaluation included probing depth (PD), assessed with a periodontal probe (PCP-UNC 15, Hu-Friedy, Chicago, IL, USA) to determine the presence and extent of periodontal pockets. From these data, the mean PD (MPD) and the number of pockets with PD ≥ 5 mm per patient (NPP5), indicative of sites with severe tissue involvement [32], were recorded. Additional parameters included the mean clinical attachment level (MAL), the full-mouth plaque score (FMPS), which measures the percentage of total tooth surfaces with visible plaque [33], and the full-mouth bleeding score (FMBS), representing the percentage of surfaces that bled upon probing [34].

These clinical parameters, in conjunction with radiographic evaluations, were used to formulate an accurate periodontal diagnosis in accordance with current classification criteria [8, 30]. The second phase of the study consisted of a 3-month period of NSPT, aimed at controlling and reducing the bacterial biofilm and calculus deposits, in accordance with EFP guidelines.

This therapeutic intervention was carried out exclusively on G2 patients [17]. Upon completion of this phase, corresponding to time point 1 (TP1), patients underwent a follow-up visit that included a new comprehensive periodontal assessment to evaluate the clinical response to treatment.

### 2.4. Saliva Sample Collection

Saliva samples were collected in the morning hours at TP0 from both G1 and G2 groups, and at TP1 only from G2 patients, using SalivaBio salivary oral swab (SOS, Salimetrics, State College, PA, USA).

Subjects were required to avoid eating, drinking, brushing teeth, and using mouthwash for at least 30 min prior to the test. These precautions were adopted to reduce interindividual variability in salivary flow and composition due to external factors, such as food and drink intake, oral hygiene products, and diurnal fluctuations. The salivary collection was administered to the participants by a trained periodontist (CB), following the manufacturer's instructions through the following steps: (a) a 30 s rinse with water, (b) a 60 s wait after the rinse, (c) intraoral positioning of proprietary and sterile salivary rollers, and (d) collection of rollers in proprietary salivary storage tubes. The SOS were placed intraorally for 1–2 min and then were centrifuged at 1500 × *g* for 15 min at +4°C (Centrifuge 5430 R, Eppendorf, Milan, Italy). The obtained samples were aliquoted into sterile Eppendorf tubes and stored at −80°C until subsequent analysis.

### 2.5. Extraction and Quantification of Salivary Proteins

Salivary proteins were extracted according to the precipitation protocol described by Jessie et al. [35]. In brief, 200 μL of saliva from both G1 and G2 groups were thawed and mixed with an equal volume of precipitation solution containing 90% acetone, 20% TCA (w/v), and 20 mM DTT, then incubated overnight at −20 °C. Samples were subsequently centrifuged at 15,000 rpm for 30 min at +4°C, and the resulting protein pellets were washed sequentially, first with a solution of 90% acetone and 20 mM DTT (wash solution 1), then with 80% acetone and 10 mM DTT (wash solution 2). After washing, the pellets were air-dried to eliminate residual acetone and resuspended in 30 μL of rehydration buffer, composed of 6 M urea, 2 M thiourea, 4% CHAPS, 25 mM DTT, and 0.2% ampholine.

Protein concentrations in the extracts were determined using the Bradford assay, with BSA as the standard and the Bio-Rad Protein Assay Dye Reagent. Absorbance (optical density, OD) was measured at 595 nm using a microplate reader (Multiskan FC, Thermo Fisher Scientific, Waltham, MA, USA).

### 2.6. Protein Separation by Electrophoresis

Three pools for each group (G1-TP0, G2-TP0, and G2-TP1) were prepared by combining multiple salivary extracts from the corresponding groups. Protein separation for each pool was performed under denaturing conditions using SDS–PAGE. Protein extracts, corresponding to 20 µg per sample, were denatured in Laemmli sample buffer supplemented with 2-mercaptoethanol, by incubation at +95 °C in a Thermomixer Comfort (Eppendorf, Milan, Italy). Protein separation was carried out on precast Bolt 4%–12% Bis-Tris Plus gels, using MES SDS–PAGE bolt running buffer, applying a constant voltage of 200 V. After separation, the gels were stained overnight with coomassie blue G-250 and subsequently destained with 5% glacial acetic acid. Gel images were acquired by a calibrated GS-800 densitometer (Bio-Rad, Hercules, CA, USA), which digitized the biological signals, that were then analyzed using the Quantity One 1-D image analysis software (v. 4.6.7, Bio-Rad Laboratories, Hercules, CA, USA), allowing for the detection of differentially expressed protein bands between healthy controls and patients with Pd.

### 2.7. LC–MS/MS Analysis

Differentially expressed protein bands were excised from the gel and processed using an “in-gel digestion” protocol [36]. Initially, the bands were destained with ACN, followed by protein reduction with DTT at +56°C, and subsequently alkylation with 55 mM iodoacetamide. Proteins were then digested with Trypsin Gold, MS grade. Peptides were extracted with a solution of 1%TFA and 50% ACN. Mass spectrometric analysis was performed by a nano UHPLC-MS system, specifically the Exploris 480 (Thermo Fisher Scientific, Reinach, Switzerland), which integrates a Nano UHPLC Ultimate 3000 System with the Exploris 480 Hybrid Quadrupole-Orbitrap Mass Spectrometer. MS data were processed using MASCOT MS/MS Ion search and the SwissProt database.

### 2.8. Quantification of cf-mtDNA and Bacterial DNA

cf-mtDNA and bacterial DNA have been quantified as previously described [37, 38]. Briefly, total DNA was extracted from 200 μL of saliva using the Qiagen DNAeasy blood kit (Qiagen, Hilden, Germany), and the amount of DNA was quantified using the NanoDrop ND-1000 (Thermo Fisher Scientific). MtDNA was quantified by droplet digital PCR (ddPCR) on a Bio-Rad QX200 ddPCR droplet system, and the reaction mixture of ddPCR had this composition in 20 μL of the final volume: 10 ng of DNA samples, 2x ddPCR Supermix for Probes (Bio-Rad Laboratories), and 1.1 μL of a validated mt-ND2 Prime PCR assay (Bio-Rad Laboratories). The thermal protocol conditions were: +95°C for 10 min, followed by 40 cycles of +94°C for 30 s, and 55°C for 1 min, then +98°C for 10 min. Droplet reading was performed on a QX200 ddPCR droplet reader (Bio-Rad Laboratories). Analysis was performed using QuantaSoft Analysis software (version 1.7.4.0917, Bio-Rad Laboratories).

Bacterial DNA burden was quantified by real-time PCR, using a mix of 10 ng of DNA samples, 5 μL of SsoAdvanced Universal SYBR Green Supermix (Bio-Rad Laboratories), 0.50 μL of the well-conserved ribosomal 16S RNA bacterial subunit (16S rDNA), and 3.5 μL of H2O water DNase/RNase free, in 10 μL final volume. The thermal protocol conditions were: +98°C for 3 min, followed by 49 cycles of +95°C for 10 s, and +60°C for 30 s, then +65°C for 5 s, +95°C for 5 s, and +16°C for 30 s.

### 2.9. Data Processing

The intensity of protein signals was calculated as OD for each band by the Quantity One 1-D image analysis software. Before protein band comparison, a filter selection process was applied on gel images (filter dimensions range from 3 × 3 pixels), to uniformly reduce or remove outlier background noise or other factors interfering with the data quantification analysis, while preserving data accuracy. The amount of each detected band was measured by its intensity, expressed as a percentage of the total signal intensity of all the defined bands in the lane, excluding the areas between bands (relative quantity calculation method, % of bands in lane).

The MS data were analyzed using the MASCOT search engine (www.matrixscience.com). Searches were conducted against the SwissProt database for peptide sequences, and cRAP for contaminants, with the following parameters: human taxonomy, trypsin as proteolytic enzyme (allowing one missed cleavage), carbamidomethylation of cysteine (fixed modification), oxidation of methionine and deamidation of asparagine/glutamine (variable modifications), mass tolerances of 10 ppm for precursor ions, and 0.05 Da for product ions, false discovery rate below 1%. Protein identifications were performed in duplicate, using bands excised from different pools for each group.

### 2.10. Statistics

Data were expressed as means ± standard deviation (SD) to describe the sample distribution.

Comparisons between clinical data were made using a parametric *t*-test or analysis of variance, followed by the Student–Newman–Keuls test for multiple comparisons, depending on whether the data were from two or three groups [39]. The paired samples *t*-test was used to compare two data sets obtained before and after treatment. For all measured variables, the null hypothesis (H₀) of no difference among groups was rejected at a significance level of *p* < 0.05. Protein concentration differences between G1 and G2 were analyzed using the independent Student's *t*-test, whereas comparisons between G2 at TP0 and TP1 were evaluated using the paired *t*-test. A *p*-value < 0.05 was considered statistically significant.

## 3. Results

### 3.1. Descriptive Clinical Analysis

The study population comprised 26 nonsmoking patients. Group G1 consisted of 16 periodontally healthy subjects with a mean age of 24.9 ± 4.5 years (range 18–32 years). All patients in this group had a full complement of 28 teeth, excluding third molars, and were free from both gingivitis and Pd. They exhibited no periodontal pockets, and none of the patients had an FMBS ≥ 10%.

Group G2 included 10 nonsmoking patients with a mean age of 52.2 ± 8.3 years (range 43–66 years). Patients in this group had an average of 25.6 ± 4.3 teeth (excluding third molars). All G2 patients presented Pd stage III/IV (5 patients in stage 3 and 5 in stage 4). On average, these patients exhibited 190 periodontal pockets with a PD ≥5 mm per patient, with a mean of 19 ± 12.8 such pockets (NPP5). Periodontal indices were measured at time 0 (TP0) in all patients (G1 and G2) and after NSPT (TP1) in G2 patients only. Clinical data are shown in Table 1.

G1 showed significantly lower periodontal indices than G2, both at TP0 and after NSPT (G2-TP1). G2 exhibited significantly higher baseline FMPS and FMBS values compared to G1. These values decreased significantly after NSPT (G2-TP1). Similarly, the periodontal parameters MPD and MAL, which were significantly higher in G2-TP0 compared to G1-TP0, showed a significant decrease after NSPT (G2 at TP1).

NSPT effectively reduced most periodontal pockets. The extent of this reduction was directly correlated with the baseline Pd value (TP0). On average, a reduction of 0.7 mm was observed, increasing to 2.4 mm for deeper pockets (≥5 mm), with most of these reduced to depths of less than 5 mm. The overall pocket closure rate was approximately 70%.

### 3.2. Quantification of Salivary Proteins and Proteomic Analysis

At baseline (TP0), the mean protein concentration in salivary extracts was significantly higher in G2 compared G1, with values of 3.05 μg/μL ± 1.17 SD vs. 2.13 μg/μL± 0.89 SD, respectively (*p*-value = 0.032). NSPT resulted in a significant reduction in salivary protein concentration in the periodontal patients, from 3.05 μg/μL ± 1.17 SD (G2-TP0) to 2.12 μg/μL ± 0.69 SD (G2-TP1) (*p*-value = 0.015).

**Figure 1:**
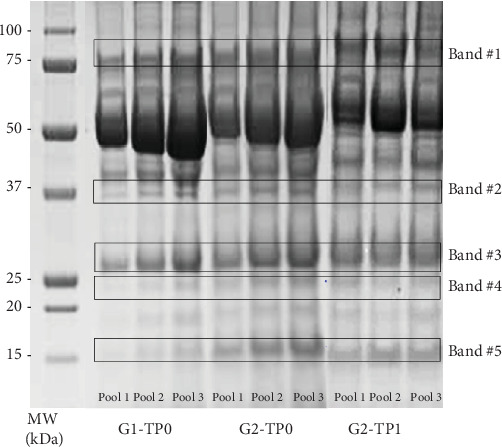
Representative 1-DE gel image. Lane 1: molecular weight (MW) marker expressed in kilodaltons (kDa); lanes 2–4: pool 1, pool 2, and pool 3 from G1-TP0; lanes 5–7: pool 1, pool 2, and pool 3 from G2-TP0; lanes 8–10: pool 1, pool 2, and pool 3 from G2-TP1. Differentially expressed bands are highlighted with squares.

SDS–PAGE of the pooled salivary proteins from each group was performed in triplicate. Densitometric analysis of the protein bands identified 5 bands significantly different (*p*-value < 0.05) between healthy and pathological saliva pool (Figure 1).

The relative quantity in percentage of the bands is reported in Table 2 (expressed as mean ± SD). The same table reports the *p*-values obtained from the comparison between G1 and G2 at baseline (G1-TP0 vs. G2-TP0) and between G2-TP0 and G2-TP1.

**Figure 2:**
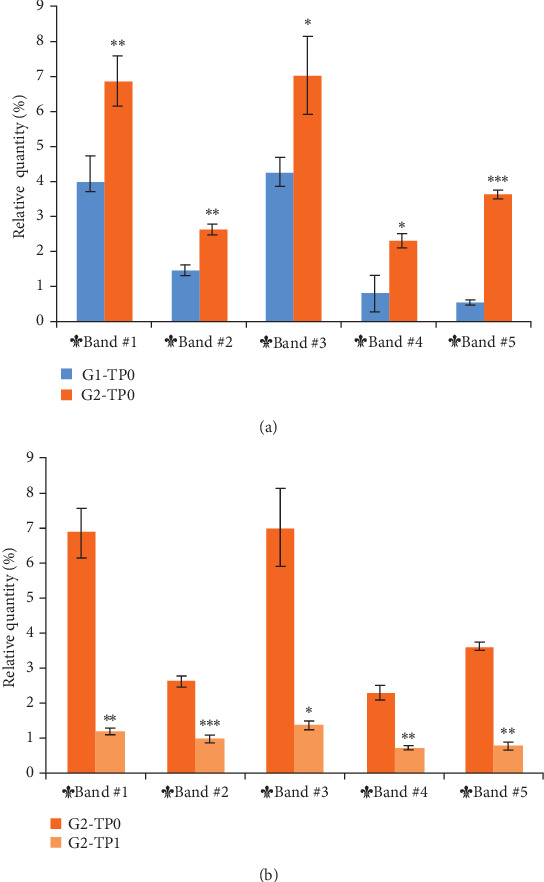
Differential protein band intensities in healthy subjects (G1) and Pd patients (G2), before (TP0) and after treatment (TP1). (A) Band intensities in G1 and G2 before treatment (G1-TP0 and G2-TP0). All five bands were significantly more abundant in Pd patients. (B) Band intensities in Pd patients before (G2-TP0) and after NSPT (G2-TP1), showing a significant reduction following treatment. Band intensities (%) are reported as mean ± SD. *p*-Values were calculated using the independent Student's *t*-test for panel (A), and the paired Student's *t*-test for panel (B) (*⁣*^*∗*^*p* < 0.05; *⁣*^*∗∗*^*p* < 0.01; *⁣*^*∗∗∗*^*p* < 0.001).

At baseline, all five bands were significantly elevated in Pd patients (G2) compared to periodontally healthy subjects (G1) (Figure 2A). Following NSPT, a significant reduction in the intensity of these bands was observed in G2-TP1 (Figure 2B).

### 3.3. Protein Identification by LC–MS/MS

The protein digest obtained from bands differentially expressed between G1 and G2 was analyzed by LC–MS/MS, resulting in the identification of 66 differentially expressed proteins (molecular weight [MW], ranging between 13 and 86 kDa), all upregulated in the salivary proteome of Pd patients before NSPT. The identified proteins and MS data are shown in Table 3.

**Figure 3:**
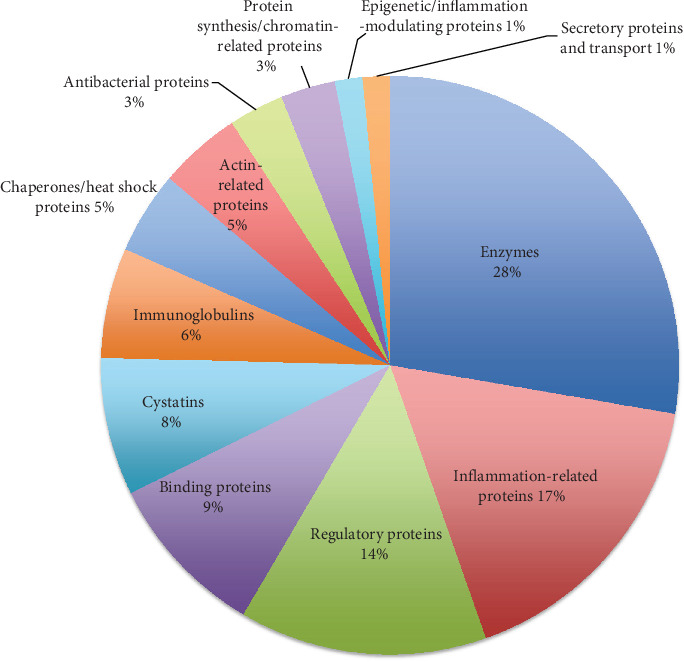
Functional clusters of salivary proteins detected in Pd patients before NSPT.

These proteins were grouped into functional clusters based on their primary roles. The largest cluster comprised enzymes (28%), followed by inflammation-related proteins (17%), regulatory proteins (14%), and binding proteins (9%). Other groups included cystatins (8%), immunoglobulins (6%), chaperones/heat shock proteins (5%), actin-related proteins (5%), antibacterial proteins (3%), protein synthesis/chromatin-related proteins (3%), epigenetic/inflammation-modulating proteins (1%), and secretory/transport proteins (1%) (Figure 3).

The identified proteins were classified into seven groups based on their potential role in Pd. These groups included proteins involved in the inflammatory and immune response, oxidative stress and redox metabolism, tissue degradation and extracellular matrix (ECM) remodeling, cell regulation and signaling, antimicrobial and defense functions, protein synthesis and energy metabolism, and secretion and transport (Table 4). However, this classification is not rigid, as some proteins may belong to multiple groups.

### 3.4. cf-MtDNA and Bacterial DNA Analysis

**Figure 4:**
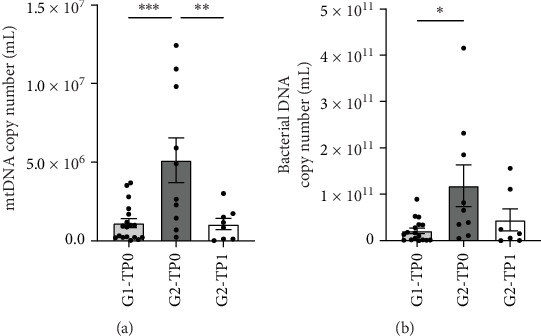
Mitochondrial DNA and bacterial DNA levels of saliva from Pd patients (G2) and periodontally healthy subjects (G1). (A) Amount of mtDNA in saliva from G1 and G2 at baseline (TP0) and after NSPT (TP1). Data are expressed as number of copies of mtDNA per mL of saliva and presented as mean ± SEM. (B) Bacterial DNA copies present in saliva between G1 and G2 at baseline (TP0), and from G2 after 3 months of treatment (TP1). Data are expressed as the number of bacterial DNA copies per mL of saliva and presented as mean ± SEM. *⁣*^*∗*^*p* < 0.05; *⁣*^*∗∗*^*p* < 0.01; *⁣*^*∗∗∗*^*p* < 0.0001.

We monitored the amount of cf-mtDNA present in saliva, as mtDNA is released extracellularly after cell death, and can act as damage-associated molecular pattern (DAMP) and promote inflammation [37, 38]. As shown in Figure 4A, at baseline, cf-mtDNA level was significantly higher in patients with Pd than in healthy controls. After NSPT, cf-mtDNA levels were significantly reduced in patients with Pd and reached levels like controls. The same trend can be observed for the bacterial DNA, with a significant increase in patients with Pd at baseline (Figure 4B), compared to healthy controls, followed by a reduction after 3 months of NSPT, although such reduction does not reach statistical significance.

## 4. Discussion

Pd is a chronic immune-inflammatory disease caused by oral microbiota infections, resulting in the destruction of tooth-supporting structures. This pathological process releases inflammatory markers and tissue degradation products into gingival crevicular fluid (GCF) and saliva, positioning this latter as a promising diagnostic fluid for identifying biomarkers related to the pathogenesis and progression of Pd [40, 41]. Diagnosis and prognosis of Pd currently rely heavily on subjective clinical assessments and require the expertise of trained periodontists. The present study aimed to identify diagnostic and predictive biomarkers of Pd, with the goal of improving understanding of its pathogenesis and facilitating an early, unbiased, molecular-based diagnosis prior to the onset of clinical periodontal damage. This objective was pursued by comparing the salivary proteome of periodontally healthy subjects (G1 group) and patients with severe Pd (G2 group), both at baseline (G1-TP0 vs. G2-TP0) and 3 months after NSPT (G1-TP0 vs. G2-TP1).

Bacterial DNA and mtDNA analysis showed that the saliva of Pd patients is characterized by a strong proinflammatory profile, presumably sustained by a high bacterial load and the presence of proinflammatory molecules, in part released actively by immune cells and in part as a result of cell death. This inflammatory environment, only partially resolved by NSPT, is highlighted by the elevated levels of bacterial DNA observed in patients with Pd at baseline, which decreased to levels comparable to those of patients without Pd following treatment. The higher levels of cf-mtDNA detected in G2-TP0 patients further suggest the presence of a chronic inflammatory state, likely associated with ongoing necrosis, resulting in the release of cf-mtDNA into the extracellular microenvironment. Since cf-mtDNA functions as a DAMP, its abundance may contribute to sustaining the proinflammatory milieu characteristic of Pd.

It is recognized that cf-mtDNA is not exclusively derived from periodontal tissues and may be elevated in a variety of inflammatory or degenerative conditions, both local and systemic. However, in our study, cf-mtDNA levels were assessed in conjunction with clinical periodontal parameters and localized sampling, which strengthens the association between cf-mtDNA and periodontal tissue damage. Moreover, the observed correlations between cf-mtDNA concentrations and specific indicators of periodontal inflammation support its relevance in this context. Nonetheless, it is known that cf-mtDNA is a general marker of cellular stress and damage, and its specificity for periodontal disease remains a limitation. Future studies should include appropriate control groups with other oral or systemic inflammatory conditions to better delineate the diagnostic specificity of cf-mtDNA in periodontal pathology.

Proteomic analysis revealed the upregulation of salivary proteins in Pd patients, mainly involved in immune-inflammatory responses, tissue destruction and repair, oxidative stress, bacterial defense, and cellular adaptation. Their overexpression underscores the centrality of inflammation in Pd, where a dysregulated immune response to microbial stimuli leads to tissue damage rather than resolution.

The identified proteins were found to be closely associated with key pathophysiological mechanisms of Pd (Table 4), supporting their potential as diagnostic or therapeutic targets.

Proteins related to inflammation and immune response play a critical role in Pd by activating immune defenses, modulating oxidative stress, and regulating inflammatory mediators. Their overexpression highlights how an excessive immune response in Pd contributes to tissue injury instead of promoting healing. Immunoglobulins, and particularly IgA, are secreted into mucosal fluids, including saliva, where they mediate bacterial recognition and neutralization. They are frequently detected in periodontal lesions and have been associated with disease severity [42, 43]. Consistent with these known functions, we observed increased levels of Ig heavy constant alpha 1 (heavy chain of IgA) and polymeric immunoglobulin receptor. Similar overexpression patterns were reported in our prior proteomic studies conducted in GCF and periodontal pocket tissue [44–46].

Proinflammatory cytokines, such as interleukin-18 (IL-18), are key mediators in Pd [42]. Proteomic analysis revealed elevated levels of IL-18 in patients with Pd, where it contributes to tissue destruction. Higher concentrations of IL-18 have been detected in GCF of Pd patients compared to those with gingivitis, supporting its role in amplifying the inflammatory response [47]. IL-18 also activates immune cells and promotes the production of additional cytokines, making it a potential therapeutic target in the management of Pd.

Other inflammatory proteins included S100-A9, which forms the calprotectin complex with S100A8 to mediate antimicrobial responses and acute inflammation [48, 49], and peroxiredoxin-1, an antioxidant enzyme that reduces peroxides and oxidative stress [50]. Both were also found to be upregulated in our previous study of periodontal tissue [44].

Proteins linked to oxidative stress and redox metabolism, such as heat shock protein HSP 90-beta and HSP beta-1, protect against oxidative damage caused by chronic inflammation and regulate free radical metabolism to prevent tissue destruction. HSP 90-beta helps stabilize proteins damaged by oxidative stress and prevents protein aggregation. It interacts with other proteins involved in the oxidative stress response, including antioxidant enzymes and transcription factors, and some studies suggest that HSP 90-beta may be regulated in chronic inflammatory conditions, such as Pd, contributing to cell survival in stressful environments [51]. HSP beta-1 in Pd plays a dual role: it protects cells from oxidative and inflammatory damage, but it may promote the survival of inflammatory cells, contributing to the chronicity of the disease. Its exact contribution depends on the specific context of the immune response and the severity of Pd. The overexpression of this protein was also detected in our previous proteomic analysis of periodontal pocket tissue [44]. Mitochondrial superoxide dismutase (Mn) (SOD2) is an antioxidant enzyme localized in mitochondria that converts superoxide anion, a toxic byproduct of mitochondrial respiration, into hydrogen peroxide and molecular oxygen, thereby reducing oxidative damage [52, 53]. As SOD2 is normally confined within mitochondria, its higher levels found in saliva suggest a release from the organelle into the extracellular space, typically observed in necrotic cells, and is in full accordance with the increase of cf-mtDNA found in the saliva of Pd patients.

Several proteins engaged in tissue degradation and remodeling of ECM were found overexpressed in Pd. They have a crucial role in tissue repair and regulation of fibroblast migration and activation; however, their dysregulation contributes to excessive tissue degradation and exacerbation of inflammation. Cathepsin L is a lysosomal cysteine protease that degrades the ECM, enhances osteoclast-mediated bone resorption, and amplifies inflammation. Its increased activity is directly associated with tissue destruction and Pd progression [54]. Gelsolin facilitates immune cell migration and tissue remodeling. Its dysregulation impairs immune responses and exacerbates tissue damage [55]. Annexin A2 (ANXA2) is crucial for inflammation modulation and periodontal tissue regeneration. It has been implicated in the inflammatory response of Pd [56] and promotes osteogenic differentiation while preventing senescence in periodontal ligament cells under high glucose conditions, suggesting a protective role in diabetic patients [57]. ANXA2 has also been found to be upregulated in our previous proteomic studies in periodontal pocket tissue, supporting its potential as a diagnostic biomarker for Pd [44, 46, 58]. Profilin-1 plays a role in actin polymerization, cell migration, and wound healing. Altered activity has been linked to inflammation and tissue breakdown [59]. Neutrophil gelatinase-associated lipocalin (NGAL) is both an inflammatory protein and a key regulator of ECM degradation [60]. It forms complexes with MMP-9, protecting it from degradation and enhancing enzymatic activity, which promotes tissue remodeling and matrix breakdown, particularly in chronic inflammatory diseases like Pd [61]. Proteasome plays a vital role in immune regulation (inflammatory-regulatory proteins) by processing antigens for MHC class I presentation, supporting lymphocyte survival, and modulating cytokine production [62]. Additionally, proteasome subunit beta type-2/3 is involved in intracellular protein degradation, playing a role in ECM integrity and cellular responses during tissue remodeling. Its function suggests a potential role in Pd progression [63]. Cystatins regulate inflammation and prevent excessive tissue degradation. Their dysregulation contributes to periodontal and bone destruction by enhancing osteoclastic activity [64, 65].

Cellular regulation and signaling proteins control cellular responses to inflammation and oxidative stress, as well as cell proliferation and migration during gingival regeneration. Several Rab proteins (Rab-11A, Rab-7A, Rab-14, Rab-1A, Rab-1B, and Rab-10) regulate intracellular trafficking, endocytosis, and exocytosis, which are essential for immune responses and tissue remodeling [66–68]. Although their specific role in Pd is not fully understood, alterations in vesicular trafficking may affect cytokine secretion and inflammatory mediator release, contributing to disease progression. Transforming protein RhoA, a small GTPase, regulates cytoskeletal dynamics, cell migration, and inflammatory signaling [69]. Serine/threonine-protein phosphatase PP1-alpha is involved in cell division, glycogen metabolism, muscle contractility, and protein synthesis, impacting inflammation, and apoptosis (uniprot.org). 14-3-3 proteins, including the zeta/delta isoform, serve as scaffold proteins in signaling pathways regulating inflammation, apoptosis, and cell survival [70]. Their upregulation in periodontal pocket tissue, as reported in our previous work, suggests a potential role as diagnostic markers for Pd [44].

Antimicrobial proteins inhibit pathogenic bacteria in the oral biofilm and help maintain a balanced microbiome. Lysozyme C targets periodontal pathogens, reflecting an immune response against bacterial biofilms. BPI fold-containing family B Member 2 functions as both an inflammatory and antimicrobial protein. It plays a role in innate immunity by modulating inflammation and protecting against gram-negative bacteria, supporting bacterial clearance [71].

Proteins involved in protein synthesis and energy metabolism provide energy to immune and stromal cells during inflammation and regulate gene expression in the inflammatory response. Glyceraldehyde-3-phosphate dehydrogenase (GAPDH) is a key enzyme in glycolysis but also influences inflammation by regulating proinflammatory gene transcription, reactive oxygen species (ROS) production, and apoptosis in immune cells. Notably, it stabilizes cytokine mRNAs (e.g., TNF-α, IL-6, and IFN-γ), modulates ROS production in macrophages, and promotes apoptosis under oxidative stress, contributing to tissue loss in Pd [72, 73]. The overexpression of GAPDH previously found in periodontal pocket tissue [44, 46] and GCF [45] supports its involvement in Pd pathogenesis.

Finally, transport and secretion-related proteins, including fatty acid-binding protein 5 (FABP5), contribute to the regulation of lipid metabolism and inflammation via PPAR-γ signaling pathways. High FABP5 levels are linked to increased immune cell infiltration and bacterial infection response, both crucial in periodontal tissue destruction [74, 75]. FABP5 has been found to be upregulated in GCF [76] and inflamed periodontal pocket tissue [44], underscoring its potential utility as a diagnostic biomarker for Pd.

Following NSPT, the levels of several proteins found upregulated at baseline in G2 were significantly decreased. These reductions paralleled clinical improvement, confirming the therapy's effectiveness in counteracting inflammation and oxidative stress.

In summary, salivary proteomic analysis reveals a robust association between severe Pd and the overexpression of proteins involved in inflammation, oxidative stress, and tissue damage. Some of these proteins were previously found in our proteomic studies conducted in GCF and periodontal pocket tissue from patients with severe Pd, reinforcing their relevance.

It should be noted that this is a pilot exploratory study, so these preliminary findings will need to be validated in future works, involving larger and independent patient cohorts and confirming the key proteins through orthogonal methods, such as ELISA tests and Western Blot.

## 5. Conclusions

The potential of salivary proteins, cf-mtDNA,and bacterial DNA as biomarkers for periodontal disease management is promising, particularly due to the noninvasive nature of saliva collection and the possibility for repeated, longitudinal sampling. However, several challenges must be addressed before these biomarkers can be integrated into routine clinical practice. These include the need for standardized protocols for saliva collection, processing, and DNA quantification to ensure reproducibility across settings. Additionally, the development of rapid, cost-effective, and user-friendly assays is essential for clinical adoption. Importantly, further longitudinal studies are required to validate the diagnostic and prognostic utility of these potential biomarkers, establish reference ranges, and assess their performance in diverse patient populations and in the presence of comorbid conditions. Addressing these factors will be critical to translating these molecular markers into practical tools for advanced treatment strategies and personalized periodontal care.

## Figures and Tables

**Table 1:** Main clinical features of G1 patients (controls) and G2 patients at TP0 and TP1.

Evaluated parameters	G1	G2	
	TP0	TP0	TP1
*N* (females)	16 (8)	10 (6)	
Age (years)	24.9 ± 4.5	52.2 ± 8.3^a^	
FMPS (%)	8.12 ± 6.03	41.10 ± 22.20^a^	20.72 ± 9.68^b^
FMBS (%)	3.62 ± 3.12	33.80 ± 15.34^a^	16.90 ± 10.82^b^
MPD (mm)	1.24 ± 0.15 mm	2.63 ± 0.65	1.94 ± 0.42^b^
MAL (mm)	1.22 ± 0.14 mm	3.56 ± 0.75	2.95 ± 0.67^b^
NPP5	0	19.00 ± 12.76	7.30 ± 11.12^b^

*Note:* Data were expressed as means ± SD. Parameters significantly different between *⁣*^a^G1–TP0 vs. G2–TP0 and ^b^G2–TP1 vs. G1–TP0.NPP5 = number of periodontal pockets.

Abbreviations: FMBS, full-mouth bleeding score; FMPS, full-mouth plaque score; MAL, mean attachment level; MPD, mean probing depth.

**Table 2:** Relative quantity of proteins bands differentially expressed in saliva.

Sample group	Relative quantity (%)				
	Band #1	Band #2	Band #3	Band #4	Band #5
G1-TP0	3.97 ± 0.76	1.47 ± 0.15	4.27 ± 0.40	0.80 ± 0.52	0.53 ± 0.06
G2-TP0	6.87 ± 0.71	2.63 ± 0.15	7.03 ± 1.12	2.30 ± 0.20	3.63 ± 0.12
G2-TP1	1.20 ± 0.10	0.98 ± 0.10	1.37 ± 0.12	0.73 ± 0.06	1.37 ± 0.12
Comparison					
G2-TP0 vs. G1-TP0	*p*=0.009	*p*=0.001	*p*=0.016	*p*=0.010	*p*=2 × 10^−6^
G2-TP0 vs. G2-TP1	*p*=0.006	*p*=0.0003	*p*=0.014	*p*=0.006	*p*=0.001

*Note:* Band numbers correspond to those reported in Figure 1. Relative quantity (%) of each significant band is expressed as mean ± SD of three replicates. *p*-Values were obtained using Student's *t*-test (significance threshold: *p* < 0.05).

**Table 3:** Differentially expressed proteins between G1 and G2 detected by LC–MS/MS.

Entry name^a^	Primary protein name^b^	Acc. No.^c^	Score^d^	Mass^e^	Match^f^	Seq.^g^	emPAI^h^	%cov.^i^	Gene name^j^
Band #1									
PIGR_HUMAN	Polymeric immunoglobulin receptor	P01833	6625	84,429	255	29	8.38	41	PIGR
GELS_HUMAN	Gelsolin	P06396	768	86,043	34	21	2.54	31	GSN
HS90B_HUMAN	Heat shock protein HSP 90-beta	P08238	117	83,554	9	7	0.49	10	HSP90AB1
Band #2									
G3P_HUMAN	Glyceraldehyde-3-phosphate dehydrogenase	P04406	4023	36,201	147	24	140.76	67	GAPDH
ANXA1_HUMAN	Annexin A1	P04083	1528	38,918	49	24	19.68	71	ANXA1
TALDO_HUMAN	Transaldolase	P37837	1409	37,688	63	23	36.58	56	TALDO1
AK1BA_HUMAN	Aldo–keto reductase family 1 member B10	O60218	1051	36,225	44	22	25.03	69	AKR1B10
LEG1H_HUMAN	Protein LEG1 homolog	Q6P5S2	740	38,244	35	14	7.13	42	LEG1
ANXA2_HUMAN	Annexin A2	P07355	711	38,808	27	17	6.89	47	ANXA2
CAH6_HUMAN	Carbonic anhydrase 6	P23280	700	35,459	28	10	2.79	40	CA6
LDHA_HUMAN	L-lactate dehydrogenase A chain	P00338	689	36,950	28	15	5.79	43	LDHA
AK1A1_HUMAN	Alcohol dehydrogenase (NADP [+])	P14550	673	36,892	27	14	5.82	48	AKR1A1
IGHA1_HUMAN	Immunoglobulin heavy constant alpha 1	P01876	614	38,486	23	10	2.41	34	IGHA1
PTGR1_HUMAN	Prostaglandin reductase 1	Q14914	566	36,075	24	13	5.24	55	PTGR1
IGHG2_HUMAN	Immunoglobulin heavy constant gamma 2	P01859	150	36,505	14	7	1.81	26	IGHG2
NAGK_HUMAN	N-acetyl-D-glucosamine kinase	Q9UJ70	489	37,694	20	12	3.48	45	NAGK
AK1C2_HUMAN	Aldo–keto reductase family 1 member C2	P52895	243	37,111	13	10	2.57	36	AKR1C2
MDHC_HUMAN	Malate dehydrogenase, cytoplasmic	P40925	273	36,631	13	11	3.12	39	MDH1
GRHPR_HUMAN	Glyoxylate reductase/hydroxypyruvate reductase	Q9UBQ7	240	36,045	13	10	2.7	35	GRHPR
CATL1_HUMAN	Cathepsin L	P07711	180	37,996	11	8	2.06	28	CTSL
PP1A_HUMAN	Serine/threonine-protein phosphatase PP1-alpha catalytic subunit	P62136	179	38,229	8	7	1.38	25	PPP1CA
Band #3									
TPIS_HUMAN	Triosephosphate isomerase	P60174	1209	31,057	42	17	16.73	74	TPI1
PRDX6_HUMAN	Peroxiredoxin-6	P30041	731	25,133	32	14	12.72	66	PRDX6
1433Z_HUMAN	14-3-3 protein zeta/delta	P63104	601	27,899	20	12	6.56	53	YWHAZ
CRIS3_HUMAN	Cysteine-rich secretory protein 3	P54108	397	28,524	22	8	7.51	51	CRISP3
PSA6_HUMAN	Proteasome subunit alpha type-6	P60900	288	27,838	12	7	2.26	32	PSMA6
PGAM1_HUMAN	Phosphoglycerate mutase 1	P18669	274	28,900	17	10	4.1	54	PGAM1
BPIA2_HUMAN	BPI fold-containing family A member 2	Q96DR5	259	27,166	15	9	3.73	34	BPIFA2
Band #4									
GSTP1_HUMAN	Glutathione S-transferase P	P09211	4174	23,569	143	16	100.81	70	GSTP1
IGK_HUMAN	Immunoglobulin kappa light chain	P0DOX7	1941	23,650	62	10	10.03	57	IGKC
PPIB_HUMAN	Peptidyl-prolyl cis–trans isomerase B	P23284	1139	23,785	41	15	23.2	57	PPIB
PEBP1_HUMAN	Phosphatidylethanolamine-binding protein 1	P30086	1124	21,158	35	11	13.49	79	PEBP1
PRDX1_HUMAN	Peroxiredoxin-1	Q06830	1043	22,324	53	19	68.19	81	PRDX1
NGAL_HUMAN	Neutrophil gelatinase-associated lipocalin	P80188	902	22,745	49	14	50.81	68	LCN2
HPRT_HUMAN	Hypoxanthine–guanine phosphoribosyltransferase	P00492	477	24,792	13	9	4.59	49	HPRT1
ZG16B_HUMAN	Zymogen granule protein 16 homolog B	Q96DA0	463	22,725	20	8	4.27	37	ZG16B
RB11A_HUMAN	Ras-related protein Rab-11A	P62491	450	24,492	21	9	4.68	42	RAB11A
RAB7A_HUMAN	Ras-related protein Rab-7A	P51149	334	23,760	15	9	5	53	RAB7A
RAB14_HUMAN	Ras-related protein Rab-14	P61106	332	24,110	14	10	6.13	60	RAB14
RAB1A_HUMAN	Ras-related protein Rab-1A	P62820	298	22,891	13	7	3.25	32	RAB1A
RAB1B_HUMAN	Ras-related protein Rab-1B	Q9H0U4	287	22,328	13	7	3.41	36	RAB1B
RAB10_HUMAN	Ras-related protein Rab-10	P61026	270	22,755	13	7	3.28	33	RAB10
BLVRB_HUMAN	Flavin reductase (NADPH)	P30043	290	22,219	11	7	3.44	50	BLVRB
RHOA_HUMAN	Transforming protein RhoA	P61586	261	22,096	12	7	3.47	43	RHOA
HSPB1_HUMAN	Heat shock protein beta-1	P04792	243	22,826	13	7	3.25	35	HSPB1
SODM_HUMAN	Superoxide dismutase (Mn), mitochondrial	P04179	218	24,906	12	7	2.79	38	SOD2
IL18_HUMAN	Interleukin-18	Q14116	216	22,597	11	7	3.31	31	IL18
PSB3_HUMAN	Proteasome subunit beta type-3	P49720	166	23,219	9	7	3.17	40	PSMB3
KCY_HUMAN	UMP-CMP kinase	P30085	158	22,436	9	6	2.54	32	CMPK1
TAGL2_HUMAN	Transgelin-2	P37802	148	22,548	7	5	1.85	28	TAGLN2
PSB2_HUMAN	Proteasome subunit beta type-2	P49721	145	22,993	11	5	2.44	28	PSMB2
Band #5									
CYTN_HUMAN	Cystatin-SN	P01037	4478	16,605	232	11	155.4	65	CST1
CYTS_HUMAN	Cystatin-S	P01036	2165	16,489	167	11	120.89	63	CST4
CYTT_HUMAN	Cystatin-SA	P09228	1544	16,719	97	10	85.64	56	CST2
PROF1_HUMAN	Profilin-1	P07737	1563	15,216	56	13	249.23	73	PFN1
FABP5_HUMAN	Fatty acid-binding protein, epidermal	Q01469	1368	15,497	46	13	66.01	70	FABP5
LYSC_HUMAN	Lysozyme C	P61626	1241	16,982	50	9	46.18	60	LYZ
RABP2_HUMAN	Cellular retinoic acid-binding protein 2	P29373	794	15,854	34	11	24.43	81	CRABP2
CYTD_HUMAN	Cystatin-D	P28325	699	16,355	23	5	6.42	49	CST5
CYTC_HUMAN	Cystatin-C	P01034	485	16,017	23	8	12.87	51	CST3
S10A9_HUMAN	Protein S100-A9	P06702	213	13,291	13	6	4.73	52	S100A9
H2B1C_HUMAN	Histone H2B type 1-C/E/F/G/I	P62807	183	13,898	5	4	2.83	35	HIST1H2BC
HINT1_HUMAN	Histidine triad nucleotide-binding protein 1	P62807	128	13,907	8	5	4.36	48	HINT1
TXD17_HUMAN	Thioredoxin domain-containing protein 17	Q9BRA2	85	14,217	7	4	2.71	32	TXNDC17
B2MG_HUMAN	Beta-2-microglobulin	P61769	69	13,820	4	3	1.74	21	B2M
RS12_HUMAN	40S ribosomal protein S12	P25398	61	14,905	4	4	2.5	31	RPS12

^a^Entry name: the protein entry name from the SwissProt database.

^b^Primary protein full name: primary protein full name from the SwissProt database.

^c^Acc. No.: primary accession number from the SwissProt.

^d^Score: the highest score obtained with MASCOT search engine.

^e^Mass: experimental protein mass (Da).

^f^Match: number of significant peptides matching the identified protein.

^g^Seq.: number of significant sequences.

^h^emPAI: exponentially modified protein abundance index.

^i^%cov.: percentage of sequence coverage.

^j^Gene name: protein gene name (from the SwissProt database).

**Table 4:** Upregulated salivary proteins in Pd grouped according to their functional role.

Inflammatory and immune response
Immunoglobulins Polymeric immunoglobulin receptor, Immunoglobulin heavy constant alpha 1, immunoglobulin heavy constant gamma 2, immunoglobulin kappa light chain
Inflammatory proteins Protein S100-A9, prostaglandin reductase 1, peroxiredoxin-1, peroxiredoxin-6, beta-2-microglobulin, interleukin-18, alcohol dehydrogenase (NADP [+]), glyoxylate reductase/hydroxypyruvate reductase

**Oxidative stress and redox metabolism**

Antioxidant and detoxifying enzymes Superoxide dismutase (Mn), mitochondrial, glutathione S-transferase P, flavin reductase (NADPH), hypoxanthine–guanine phosphoribosyltransferase, thioredoxin domain-containing protein 17, aldo–keto reductase family 1 member B10, aldo–keto reductase family 1 member C2, peptidyl-prolyl cis–trans isomerase B
Heat shock protein Heat shock protein HSP 90-beta, heat shock protein beta-1

**Tissue degradation and remodeling of the extracellular matrix (ECM)**

Degradative enzymes Cathepsin L, phosphoglycerate mutase 1, carbonic anhydrase 6
Tissue remodeling-associated proteins Gelsolin, annexin A1, annexin A2, transgelin-2, profilin-1, neutrophil gelatinase-associated lipocalin, proteasome subunit alpha type 6, proteasome subunit beta type-2, proteasome subunit beta type-3
Cystatins Cystatin-SN, cystatin-S, cystatin-SA, cystatin-D, cystatin-C

**Cell regulation and signaling/regulatory proteins**

Rab protein family Ras-related protein Rab-11A, ras-related protein Rab-7A, ras-related protein Rab-14, ras-related protein Rab-1A, ras-related protein Rab-1B, ras-related protein Rab-10 Transforming protein RhoA Serine/threonine-protein phosphatase PP1-alpha 14-3-3 protein zeta/delta Histidine triad nucleotide-binding protein 1 Protein LEG1 homolog

**Antimicrobial and defense proteins**

Lysozyme C BPI fold-containing family A member 2

**Protein synthesis and energy metabolism**

Enzymes Triosephosphate isomerase, UMP-CMP kinase, N-acetyl-D-glucosamine kinase, glyceraldehyde-3-phosphate dehydrogenase, malate dehydrogenase, cytoplasmic, transaldolase, L-lactate dehydrogenase A chain 40S ribosomal protein S12 Histone H2B type 1-C/E/F/G/I

**Secretory and transport proteins**

Fatty acid-binding protein, epidermal Phosphatidylethanolamine-binding protein 1 Cellular retinoic acid-binding protein 2 Zymogen granule protein 16 homolog B Cysteine-rich secretory protein 3

## Data Availability

The data are available upon request from the authors.

## Nomenclature

GR: Gingival recession depth
CEJ: Cemento-enamel junction
Cf-mtDNA: Cell-free mitochondrial DNA
DAMP: Damage-associated molecular pattern
ddPCR: Droplet digital PCR
ECM: Extracellular matrix
EFP: European Federation of Periodontology
FMBS: Full-mouth bleeding score
FMPS: Full-mouth plaque score
G1: Periodontally healthy individuals
G2: Patients with advanced periodontitis
GCF: Gingival crevicular fluid
LC–MS/MS: Liquid chromatography–mass spectrometry
MAL: Mean attachment level
MPD: Mean probing depth
MS: Mass spectrometry
NPP5: Number of pockets with PD ≥ 5 mm per patient
NSPT: Nonsurgical periodontal therapy
Pd: Periodontitis
PD: Probing depth
SDS–PAGE: Sodium dodecyl sulfate-polyacrylamide gel electrophoresis
SOS: Salivary oral swab
TP0: Time point 0
TP1: Time point 1.
